# High Myopia: A Pointer of an Inborn Error of Metabolism

**DOI:** 10.7759/cureus.20930

**Published:** 2022-01-04

**Authors:** Sanjay Kumar Sahu, Jyoti Ranjan Behera, Jishnu KR, Moparthi Puramjai, Ramakrushna Gudu, Arun K Dash, Amit R Rup, Sibabratta Patnaik

**Affiliations:** 1 Pediatrics, Kalinga Institute of Medical Sciences, Bhubaneswar, IND; 2 Paediatrics, Kalinga Institute of Medical Sciences, Bhubaneswar, IND

**Keywords:** cerebral sinovenous thrombosis, cystathionine beta synthase, homocystinuria, pyridoxine, csvt, ectopia lentis

## Abstract

An 11-year-old boy with marfanoid habitus and high myopia presented with multiple episodes of seizures. He was found to have arachnodactyly, hypermobile joints, ectopia lentis, cerebral venous sinus thrombosis (CVST) with very high serum methionine and homocysteine. Genetic evaluation unveiled homocystinuria due to cystathionine beta-synthase deficiency. The patient was treated with high-dose pyridoxine, methionine restricted diet, anticonvulsants, warfarin, and correction of ectopia lentis. Homocystinuria should be suspected in patients with tall stature and pathological myopia. Early treatment can prevent thromboembolic complications.

## Introduction

Homocystinuria is an inborn error of methionine metabolism due to deficiency of cystathionine-β-synthase (CBS) with an incidence of one in 200,000 to one in 350,000 live births and an even higher incidence of one in 800 live births is seen in some countries like Qatar [1]. It is inherited as an autosomal recessive trait. The spectrum of manifestations varies from being asymptomatic to severe multisystem involvement. The chief presenting features are ectopia lentis, marfanoid habitus, progressive intellectual disability, and thromboembolic phenomena [2]. We present the case of an 11-year-old boy presenting with high myopia and seizures diagnosed to have classic homocystinuria.

## Case presentation

**Figure 1:**
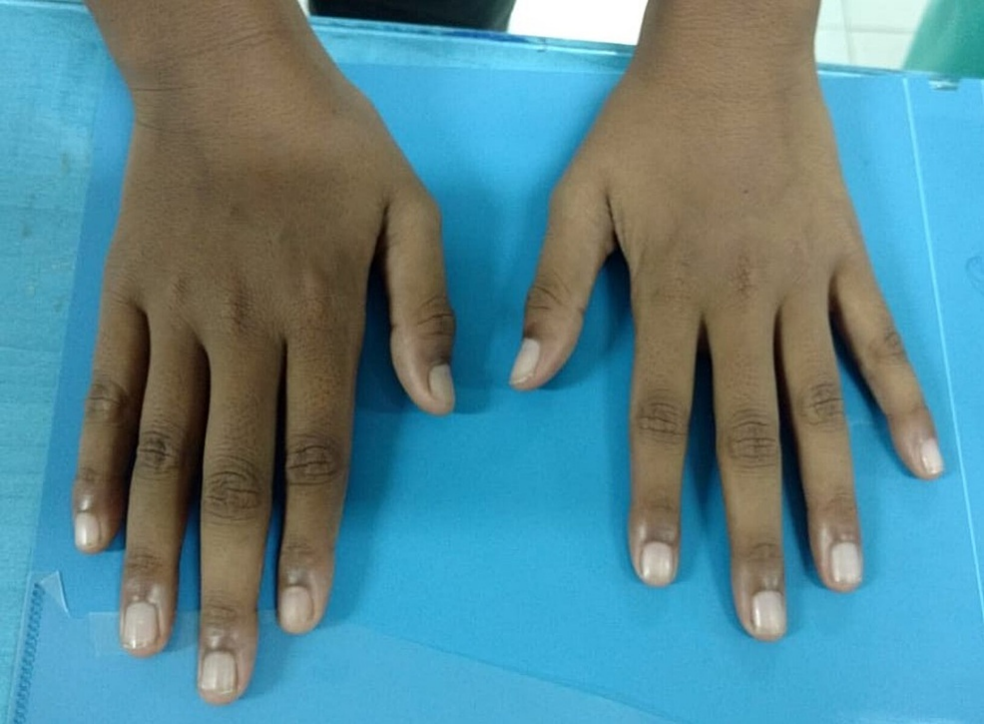
Arachnodactyly (Long, slender and curved fingers)

An 11-year-old-boy presented to the emergency department with multiple episodes of generalized seizures for one day with no history of fever, vomiting, altered sensorium, or focal neurological deficit. He was having high myopia of - 22D and poor scholastic performance. There was no past history or family history of seizure. He was born out of non-consanguineous marriage with a birth weight of 3.2 kg and an uneventful perinatal period. There was no history of sibling death or abortion. Anthropometry revealed a height of 165 cm, which was greater than +2 SD for his age, suggestive of tall stature. On examination, the child was having marfanoid habitus with hypermobility of joints and arachnodactyly (Figure 1). There were no neurocutaneous markers. Cardiac and other systemic examinations were normal.

**Figure 2:**
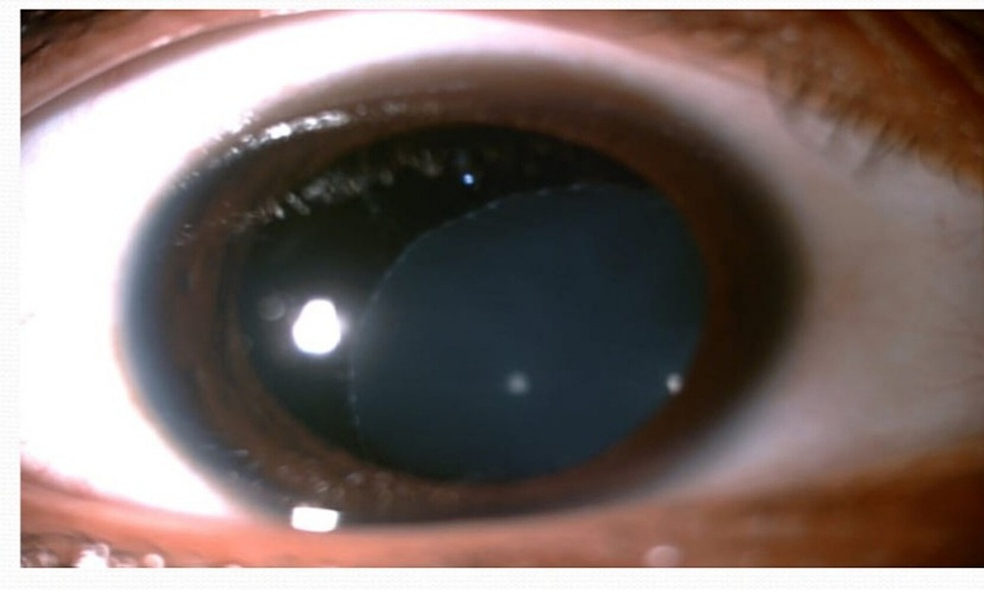
Inferonasal displacement of lens (ectopia lentis) of right eye. Similar finding was also present in left eye.

**Figure 3:**
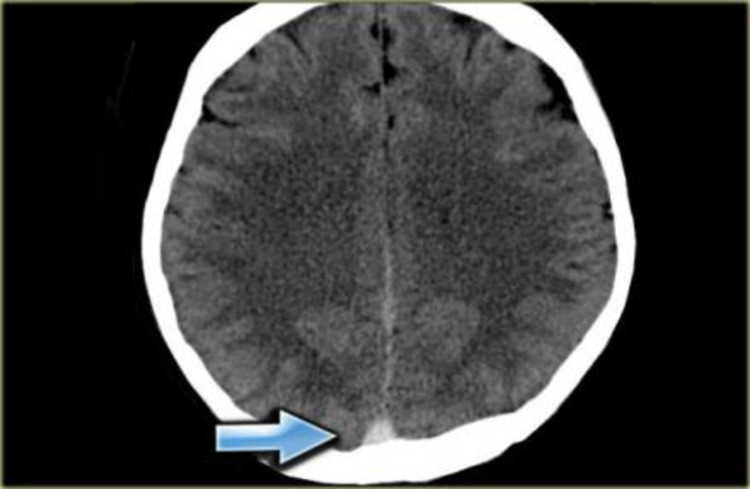
CECT brain showing CVST (arrowhead) CECT: contrast-enhanced computed tomography; CVST: cerebral venous sinus thrombosis

On investigation, complete blood counts revealed hemoglobin 12 gm/dl, total leucocyte count 7800/cu mm and total platelet count 4 lacs /cu mm. C- reactive protein was 2 mg/L, serum sodium 139 mmol/L, serum potassium 4.4 mmol/L, and serum calcium was 9 mg/dL. Ophthalmological examination was done in view of high myopia and revealed ectopia lentis in both eyes (Figure 2). Contrast-enhanced computed tomography (CECT) brain revealed cerebral venous sinus thrombosis (CVST) (Figure 3).

Homocystinuria, Marfan syndrome, and prothrombotic disorders (protein C deficiency, protein S deficiency, and antithrombin III deficiency) were considered as the differentials. On further workup, the coagulation profile was normal (PT-11.6 secs, INR - 0.9, aPTT - 26 secs). Protein C, protein S, and antithrombin III levels were within the normal limit. Echocardiography revealed no abnormality. Both serum and urinary homocysteine and methionine level were elevated. Genetic evaluation unveiled CBS deficiency confirming the diagnosis of homocystinuria. He was advised a methionine-restricted diet, a high dose of pyridoxine, and folic acid. For control of seizures, anticonvulsant was started. Low molecular weight heparin was given for CVST. Ectopia lentis was corrected surgically following which myopia dramatically improved to -10D. The child was discharged on warfarin for CVST.

**Table 1:** Value of various laboratory parameters

Parameters	Observed value	Reference range
Hemoglobin	12 gm/dl	11.1 – 14.1 gm/dl
Total Leucocyte count	7.8 ×10^3^/µL	5 – 15 ×10^3^/µL
Total RBC count	4.2 ×10^6^/µL	3.9 – 5.1×10^6^/µL
Total Platelet count	400 ×10^3^/µL	200 – 550 × 10^3^/µL
C Reactive Protein	2 mg/L	< 5 mg/L
Serum sodium	139 mmol/L	138 - 145 mmol/L
Serum potassium	4.4 mmol/L	3.4 – 4.7 mmol/L
Serum calcium	9 mg/dL	8.5 - 10.5 mg/dL
Prothrombin time	11.6 sec	10 – 15 secs
Activated partial thromboplastin time	26 sec	25 – 36 secs

On the six-month follow-up, the child was seizure-free without any thrombotic episode. Both plasma and urine homocysteine and methionine levels had decreased. He was also on regular ophthalmological follow-up. After two years of diagnosis, he was off anticonvulsant; however, scholastic performance continues to be poor.

## Discussion

The distinctive features of homocystinuria are marfanoid habitus, ectopia lentis, intellectual disability, thromboembolic phenomena, and osteoporosis [2]. Regardless of age and typical clinical features, homocystinuria should be considered in the differential diagnosis of thrombotic episodes [3]. As ectopia lentis is the hallmark and the most consistent feature, finding a dislocated lens should always raise suspicion of homocystinuria [4]. It is also found in conditions like Ehlers Danlos syndrome, Weill Marchesani syndrome, and sulfite oxidase deficiency. 

Based on clinical manifestations and biochemical findings, homocystinuria is classified into three types. Type 1 is associated with hypermethioninemia in addition to homocystinuria, which is also termed as classical variety and usually presents after three years of age. Type 2 presents early with feeding difficulty, vomiting, lethargy, hypotonia, and developmental delay with hypomethioninemia and megaloblastic anemia. Type 3 can present during the neonatal period or with chronic manifestations at a later age. Similar to type 2, these patients have hypomethioninemia but an absence of megaloblastic anemia is the differentiating feature [5].

The main aim of the treatment is to lower the plasma homocysteine level by strategies like high dose pyridoxine, folic acid, and dietary restriction of methionine along with treatment of complications like ectopia lentis and thromboembolic episodes, if present. Recent researches revealed that with plasma total homocysteine concentration below 100 μmol/L, the occurrence of thromboembolic episodes is less likely. The dose of pyridoxine is 10 mg/kg/day with 500 mg/day being the maximum dose to continue for lifelong. Adequate folate should be supplemented to all cases with monitoring of vitamin B12 [6].

For those who cannot achieve target levels of homocysteine by pyridoxine and dietary modification, betaine should be considered as an adjunctive. It decreases homocysteine content by converting homocysteine to methionine with the donation of a methyl group. To start, the dose is 100 mg/kg/day in two divided doses and then titrated according to response (weekly increased by 50 mg/kg) [6]. The maximum licensed dose is 3 grams twice daily. In all patients with CBS deficiency, total homocysteine, plasma methionine, vitamin B12, and folate should be monitored frequently. Development of new complications can be prevented by long-term treatment and with good biochemical control but it does not reverse complications those already present. Currently, newborn screening is available for homocystinuria. However, homocystinuria is not a part of routine newborn screening in India (from where the case is reported) till now and it was also not available at the time of the birth of the child. It can be diagnosed from enzyme deficiency in the extract of cells from cultured amniotic fluid and early treatment can be initiated to prevent complications [7]. As the chances of recurrence in subsequent pregnancies increase, a prenatal diagnosis should be done in all subsequent pregnancies of women who already have an affected child [7].

## Conclusions

Homocystinuria is an inborn error of methionine metabolism with many life-threatening adverse outcomes, including intellectual disability and visual impairment. Hence, the entity should be suspected in all children with high myopia, lens dislocation, marfanoid habitus, or thromboembolism. In newborns, screening for inborn errors of metabolism should include early detection of homocystinuria. Timely diagnosis is vital for preventing visual and intellectual disability and also prevents the birth of another affected child. This may improve the quality of life of the patient and ultimately helps in decreasing the disease burden of society.
